# CORO1C is Associated With Poor Prognosis and Promotes Metastasis Through PI3K/AKT Pathway in Colorectal Cancer

**DOI:** 10.3389/fmolb.2021.682594

**Published:** 2021-06-10

**Authors:** Zongxia Wang, Lizhou Jia, Yushu sun, Chunli Li, Lingli Zhang, Xiangcheng Wang, Hao Chen

**Affiliations:** ^1^ Cancer Center, Bayannur Hospital, Bayannur, China; ^2^ Department of Pathology, Wannan Medical College, Wuhu, China; ^3^ Department of Oncology, Inner Mongolia Autonomous Region Cancer Hospital, Hohhot, China; ^4^ Department of Ophthalmology, Inner Mongolia Autonomous Region People’s Hospital, Hohhot, China; ^5^ Department of Nuclear Medicine, The Affiliated Hospital of Inner Mongolia Medical University, Hohhot, China; ^6^ Key Laboratory of Inner Mongolia Autonomous Region Molecular Imaging, Inner Mongolia Medical University, Hohhot, China; ^7^ Faculty of Medical Science, Jinan University, Guangzhou, China

**Keywords:** CORO1C, colorectal cancer, prognosis, metastasis, AKT

## Abstract

Trophoblast cell surface protein 2 (Trop2) is one of the cancer-related proteins that plays a vital role in biological aggressiveness and poor prognosis of colorectal cancer (CRC). The study of the Trop2 related network is helpful for us to understand the mechanism of tumorigenesis. However, the effects of the related proteins interacting with Trop2 in CRC remain unclear. Here, we found that coronin-like actin-binding protein 1C (CORO1C) could interact with Trop2 and the expression of CORO1C in CRC tissues was higher than that in paracarcinoma tissues. The expression of CORO1C was associated with histological type, lymph node metastasis, distant metastasis, AJCC stage, venous invasion, and perineural invasion. The correlation between CORO1C expression and clinical characteristics was analyzed demonstrating that high CORO1C expression in CRC patients were associated with poor prognosis. Furthermore, CORO1C knockdown could decrease the cell proliferation, colony formation, migration and invasion *in vitro* and tumor growth *in vivo*. The underlying mechanisms were predicted by bioinformatics analysis and verified by Western blotting. We found that PI3K/AKT signaling pathway was significantly inhibited by CORO1C knockdown and the tuomr-promoting role of CORO1C was leastwise partly mediated by PI3K/AKT signaling pathway. Thus, CORO1C may be a valuable prognostic biomarker and drug target in CRC patients.

## Introduction

Colorectal cancer (CRC) is the third most common cancer worldwide, and the incidence rate and mortality of CRC are increasing year by year (Siegel et al., 2020). Metastasis continues to be the leading cause of significant clinical problems and more than 90% cancer-related mortalities (Guan, 2015). Liver is the most common site of distant relapse in CRC patients, followed by lung, hilar/perihepatic lymph nodes, and peritoneum (Jiang et al., 2021). Approximately 25% of CRC patients present distant metastasis at initial diagnosis, and 50% of them develop metastatic disease within 3 years (Vatandoust et al., 2015). Cell migration underlies malignant tumor invasion and metastasis.

Human trophoblast cell surface protein 2 (Trop2) is a cell-surface glycoprotein highly expressed in a variety of tumors, and the high expression of Trop2 protein is associated with poor survival prognosis of cancer patients (Zhao et al., 2017; Zimmers et al., 2018; Sun et al., 2020). Trop2 plays a key role in inducing epithelial-mesenchymal transition (EMT) and regulating cell migration (Li et al., 2017; Wu et al., 2017; Gu et al., 2018). Trop2 physically interacts with β-catenin which is a vital molecule of EMT, and Trop2-induced β-catenin accumulation in the nucleus accelerates gastric tumor metastasis (Zhao et al., 2019). Due to the complicated mechanisms of Trop2, it remains necessary to investigate the interacting proteins of Trop2 in the process of exploring tumorigenesis.

When it comes to the potential function of Trop2, we found that coronin-like actin-binding protein 1C (CORO1C) has interacted with it. CORO1C belongs to the highly conserved coronin family that affects the actin cytoskeleton (Liu et al., 2016). It is enriched at the leading edge of lamellipodia, as well as folds of the invasive cell membrane (Brayford et al., 2016). CORO1C regulates F-actin, Arp2/3 complex, and ADF/cofilin proteins, inhibiting actin dynamics (Chan et al., 2011). It also selectively interferes with Rac1, which is a nexus in membrane protrusion and migration regulation (Williamson et al., 2015). Furthermore, CORO1C is differentially expressed in various solid tumors, such as glioblastoma cancer (Thal et al., 2008), hepatocellular cancer (Wu et al., 2010b), breast cancer (Wang et al., 2014) and lung cancer (Mataki et al., 2015). CORO1C has been confirmed to promote cellular proliferation and metastasis through regulating cyclin D1 and vimentin in gastric cancer (Cheng et al., 2019), but little is known about the role of CORO1C in CRC.

In this study, we investigated the proteins conjugated with Trop2 and speculated that CORO1C plays a vital role in CRC metastasis. The expression of CORO1C in CRC was detected, and the associations between CORO1C and clinicopathological features of CRC patients were investigated Also, the effects of CORO1C on CRC cells and the underlying mechanisms were explored *in vitro* and *in vivo*. Our research confirmed the biological function of CORO1C in CRC cells and provided a novel insight to CRC development and progression.

## Materials and Methods

### Cell Line and Cell Culture

293T cell line and colorectal cancer cell lines SW480, SW620, COCA2, LOVO, HCT116,HT-29, and the normal colorectal epithelial cell line (NCM460) were purchased from Keygen Biotech Co., Ltd. and maintained in DMEM medium (Gibco, United States) with 10% fetal bovine serum (Gibco, United States), 50 units/ml penicillin (Gibco, United States) and 50 μg/ml streptomycin (Gibco, United States). The cell line was incubated in a moist environment with 5% CO_2_ at 37°C. AKT inhibitor (Miltefosine) was perchased (Selleck Chemicals LLC,S3056) and the used concentration was 50 μM.

### Lentivirus-Mediated Transfection for Trop2 and CORO1C Over-Expression

The lentivirus-mediated Trop2-cDNA, CORO1C-cDNA (GeneCopoeia, Guangzhou, China) inserted in the pcDNA3.1 were used to overexpress Trop2 in 293T cells. Lipofectamine 2000 Transfection Reagent (Invitrogen, United States) was utilized to perform the transfections according to the instruction.

### shRNA for CORO1C Down-Expression

The shRNA of CORO1C was designed according to the sequence of CORO1C gene (sense sequence: GGT​AGT​CAG​CTG​GGA​AAG​TCT), and BLAST test showed no homology with the coding sequence of other genes. CORO1C knockdown plasmids were synthesized by GeneCopoeia Co., Ltd. (Top strand: 5′-CAC​CGG​TAG​TCA​GCT​GGG​AAA​GTC​TCG​AAA​GAC​TTT​CCC​AGC​TGA​CTA​CC-3'. Bottom strand: 5′- AAA​AGG​TAG​TCA​GCT​GGG​AAA​GTC​TTT​CGA​GAC​TTT​CCC​AGC​TGA​CTA​CC -3′). The plasmid was transformed into *Escherichia coli* DH5α for amplification, and then extracted by EndoFree Maxi Plasmid Kit (TianGen, Beijing, China) according the instructions. Lipofectamine 2000 Transfection Reagent (Invitrogen, United States) was utilized to perform the transfections according to the instructions.

### Quantitative Polymerase Chain Reaction (qPCR) Array

Total RNA was extracted from 293T cells using Trizol reagent (#12096028, Invitrogen, United States) and reverse transcribed into cDNA using High Capacity cDNA Reverse Transcription Kits (#4374966, Applied Biosystems, United States) according to the instruction. Quantitative real-time PCR was performed using SYBR Advantage qPCR Premix (#638321, Takara, Japan) on an ABI StepOnePlus Real-Time PCR System following the steps below: 95°C for 20 s; 40 cycles of amplification at 95°C for 15 s and 56°C for 1 min; 95°C for 15 s, 60°C for 1 min, 95°C for 15 s. Following primers were used for the reaction: human Trop2 forward, 5ʹ-ACA​ACG​ATG​GCC​TCT​ACG​AC-3ʹ, and reverse, 5ʹ- GTC​CAG​GTC​TGA​GTG​GTT​GAA-3ʹ, and GAPDH forward, 5ʹ-GGA​GCG​AGA​TCC​CTC​CAA​AAT-3ʹ, and reverse, 5ʹ-GGC​TGT​TGT​CAT​ACT​TCT​CAT​GG-3ʹ. Results were normalized to GAPDH and all experiments were repeated in triplicate.

### Protein Extraction and Western Blotting

293T cells were dissolved by RIPA Lysis Buffer (#89901, Thermo, United States) in accordance with the protocol. The lysate was kept on ice for 5 min and then centrifuged at 12,000 g for 20 min. Protein samples were mixed with SDS-PAGE sample loading buffer (#P0015, Beyotime, China) and then heated to 95°C for 10 min. The protein sample was loaded onto an polyacrylamide gel for electrophoresis and then transferred to a PVDF membrane. The blots were blocked at room temperature for 2 h and then incubated with appropriate primary antibodies overnight at 4°C. After washing three times with PBST, the membrane was incubated with corresponding secondary antibodies at room temperature for 1 h. Proteins were visualized by chemiluminescent substrate (#34580, Thermo, United States) and ChemiDoc XRS + system (Bio-Rad, United States). Primary antibodies was used to detect Trop2 (1:1,000, #90540, CST, United States), CORO1C (1:500, #14749-1-AP, Proteintech, United States) and GAPDH (1:1,000, #97166, CST, United States).

### Immunoprecipitation and Mass Spectrometry

10 µg anti-Trop2 IgG (#90540, CST, United States) was incubated with 50 µL dynabeads (#20423, Thermo, United States) for 10 min with rotation at room temperature. The protein sample was incubated with dynabead-IgG complex for 10 min at room temperature with rotation. The complex was washed with PBST three times and then resuspended by glycine (50 nM, pH2.8) for 2 min with rotation. The eluate obtained from the IP experiment was incubated with 1 mmol DTT and 200 μL UA buffer (8 MUrea, 150 mM TrisHCl,pH8.0) at room temperature for 1 h. The mixture was transferred into 10 kDa ultrafiltration centrifuge tube, centrifuged for 15 min (14,000 g). The tube was added with 14,000 μL IAA (50 mM), shocked for 1 min, incubated in room temperature for 30 min, and centrifugated at 14,000 g for 10 min. Ammonium bicarbonate (300 μL, 100 mmol) was added to the tube, and the tube was centrifuged in 14, 000 g for 20 min. The tube was added with 8 μL Trypsin buffer (4 μg Trypsin) and incubated in 37°C for 16 h. The filtrate was collected by centrifugation at 14, 000 g for 10 min and detected by TripleTOF 5,600 + mass spectrometer (AB SCIEX, United States). The data was analyzed by MaxQuant 1.5.2.8 software, and then the peptides and proteins were identified by Maxquant algorithm. The filtration parameters were set as: peptide FDR ≤0.01 and protein FDR ≤0.01.

### Patients and Tissue Specimens

In this study, 581 patients with CRC diagnosed in the Affiliated Hospital of Nantong University during 2014–2018 were recruited. A total of 734 formalin-fixed, paraffin-embedded (FFPE) CC tissue samples were investigated, including CRC tissues (*n* = 581), matched paracarcinoma tissues (*n* = 117), and colonitis tissues (*n* = 36). None of the patients had been treated with radiotherapy, chemotherapy, or immunotherapy. The clinical information, including age, gender, location, histologic type, differentiation, depth of invasion, lymph node metastasis, distant metastasis, AJCC stage, venous invasion, perineural invasion, preoperative CEA, preoperative CA199, and Ki67 about the patients enrolled in this study were recorded.

### Tissue Microarrays and Immunohistochemistry

The TMAs were constructed in the Department of Pathology, Affiliated Hospital of Nantong University, Nantong, Jiangsu, China, using the Quick-Ray tissue system (UNITMA, Korea). Graded alcohol was used for deparaffinage and rehydration. 3% H_2_O_2_ was utilized to block endogenous peroxidase. Antigens were retrieved by heating in 0.01 M citrate buffer (pH 6.0). CORO1C was detected by a anti-human CORO1C rabbit polyclonal antibody (dilution 1:200) (#14749-1-AP, Proteintech, United States). Hematoxylin was used for counterstain. Staining was scored independently by two pathologists unaware of clinical characteristics. CORO1C expression was quantified according to the semi-quantitative H-score method, using staining intensity scores as follow: 0 indicated negative expression; 1 indicated weakly positive staining; 2 indicated moderately positive staining; and 3 indicated strongly positive staining. Single staining intensity score = score × the percentage of cells in the corresponding intensity × 100. Final staining scores were the sum of four staining intensity scores. The minimum possible final staining score was 0 (no staining) and maximum possible score was 300 (100% of cells with 3 staining intensity) (Zhao et al., 2016).

### CCK-8 Proliferation Assay

The cells in each group were seeded into 96-well plates with 5 × 10^3^ cell/well, and cell proliferation was detected using CCK-8 kit according to the instruction. Diluted CCK-8 reagents were added into each well at 24, 48, 72 and 96 h respectively. After incubation in 37°C for 2 h, the absorbance of cells in each group at 450 nm was detected by a microplate reader.

### Clone Formation Assay

The cells in each group were seeded into 6-well plates with 1 × 10^3^ cell/well. After incubation for 2 weeks, cells were fixed with formaldehyde for 30 min, and then stained with 0.01% crystal violet for 30 min. Cell clones were counted and photographed.

### Transwell Migration/Invasion Assay

Medium containing 10% serum was added in the lower chamber, cell suspension without serum was added in the upper chamber (containing matrigel glue for invasion assay). After incubation for 48 h, the membrane of the upper chamber was sucked and then fixed with formaldehyde for 30 min. Cells on the membrane were stained with 0.01% crystal violet for 30 min. Average values of 5 visual fields were randomly selected for statistical analysis.

### Nude Mouse Tumorigenicity Assay

HCT116-shControl and HCT116-shCORO1C were collected in the logarithmic growth phase, respectively, and 100 μL cell suspension with a density of 1 × 10^8^/ ml was prepared with RPMI 1640 medium and injected into the oxter of nude mice. The tumor size was measured with Vernier calipers every 3 days. Tumor volume = longest diameter × shortest diameter^2^/2. The protocols of animal study were approved by the laboratory animal center of Inner Mongolia Medical University.

### Gene Ontology Analysis and Gene Set Enrichment Analysis

Ualcan database (http://ualcan.path.uab.edu/) was searched, and 395 genes were identified to be closely associated with CORO1C (pearson correlation coefficient ≥0.5, data not shown). Next, we performed GO analysis and GSEA of these CORO1C-related genes by DAVID database (https://david.ncifcrf.gov/). These genes were classified into three functional groups: molecular function group, biological process group, and cellular component group. Terms in each group and signaling pathways in KEGG were selected if *p* < 0.001 and false discovery rate <0.1. Data were visualized using Sangerbox online tool (http://sangerbox.com/).

### Statistical Analysis

All statistics were analyzed by SPSS 19.0 statistical software (SPSS Inc., Chicago, IL). All the experiments were repeated three times with similar results. The correlation between CORO1C expression and clinical features was analyzed by Pearson’s χ^2^ test. The differences between the two groups were analyzed using an unpaired Student’s *t*-test. Three or more groups were compared using one-way analysis of variance (ANOVA), followed by Tukey’s multiple comparison test. Cumulative patient survival was estimated by Kaplan-Meier analysis. The survival curves were compared by log-rank test. *p* < 0.05 indicated statistical significance.

## Results

### CORO1C is Related to Colorectal Cancer Cell Migration and Invasion

**FIGURE 1:**
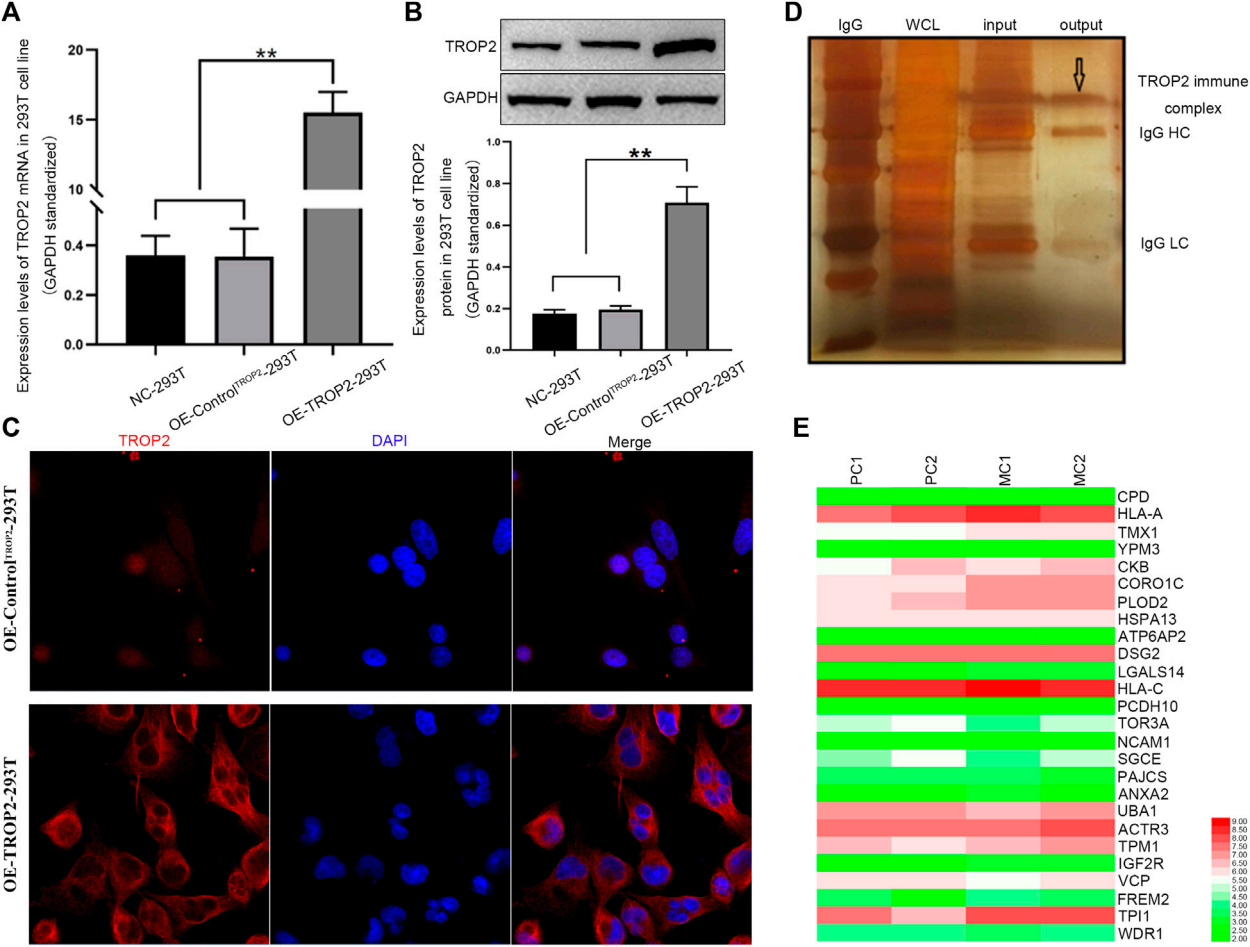
Associations between CORO1C, TROP2, and CRC metastasis. Overexpression of Trop2 in 293T cells was confirmed by **(A)** qRT-PCR, **(B)** Western blotting, and **(C)** Immunofluorescence. **(D)** Trop2 immunocomplex by Co-IP; **(E)** Heatmap of proteins interacting with Trop2 in PC and MC. The RNA-seq data were obtained from GSE28702. Red and green colors represent high and low gene expression, respectively. PC: primary CRC; MC: metastatic CRC, **p < 0.01.

To identify the proteins interacting with Trop2, we first overexpressed Trop2 in 293T cells using the lentivirus-mediated Trop2-cDNA. qPCR, Western blotting, and immunofluorescence were performed to confirm Trop2 overexpression (Figures 1A–C). Coimmunoprecipitation (Co-IP) and mass spectrometry were used to define the proteins (CPD, HLA-A, PLOD2, and CORO1C) interacting with Trop2 (Figure 1D, Supplementary Table S1). Bioinformatics analysis confirmed that CORO1C is strongly associated with invasion and metastasis (Figure 1E, Supplementary Table S2).

### CORO1C Protein was Overexpressed in Colorectal Cancer Tissues Compared to Adjacent Tissues

**FIGURE 2:**
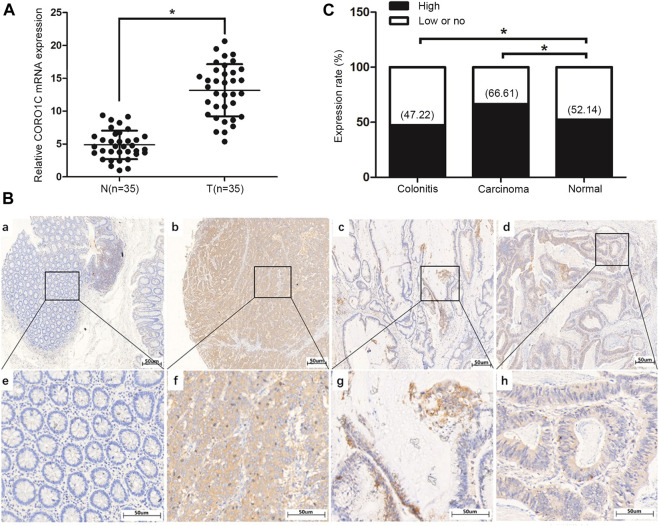
Expression of CORO1C in CRC patients. **(A)** CORO1C mRNA levels in the 35 CRC tissues. **(B)** Representative images of CORO1C protein expression in colorectal tissues: a. Negative expression of CORO1C in normal colorectal tissues; b. Positive expression of CORO1C in moderately differentiated tubular adenocarcinoma tissues; c. Positive expression of CORO1C in mucinous adenocarcinoma tissues; d. Positive expression of CORO1C in highly differentiated tubular adenocarcinoma tissues. **(C)** Positive rate of high CORO1C in CRC tissues was higher than in normal tissues, *p < 0.05.

The mRNA level of *COR O 1C* in CRC tissues was confirmed to be higher than that in normal tissues (Figure 2A). The protein subcellular localization of CORO1C, detected by IHC assay was in the cytoplasm in CRC tissues (Figure 2B). The χ-tile software program for TMA data analysis was utilized to estimate the level of CORO1C protein in CRC patients. The score between 0 and 130 was considered low or no expression, while the counts >130 were considered high expression. The CORO1C expression was classified as high or low or no. The frequency of high CORO1C expression in CRC tissues (66.61%, 387/581) was higher than that in pericarcinomatous tissue (52.14%, 61/117) (Figure 2C).

### CORO1C Overexpression was Associated With Increased Invasiveness and Metastasis in Colorectal Cancer Patients

We investigated the relationship between CORO1C expression and pathological parameters in CRC patients. The results showed that the expression of CORO1C was significantly related to the histological type (χ^2^ = 7.6419, *p* = 0.006), lymph node metastases (χ^2^ = 19.1615, *p* < 0.001), distant metastases (χ^2^ = 9.5623, *p* = 0.002), AJCC stage (χ^2^ = 17.7192, *p* = 0.001), venous invasion (χ^2^ = 22.1337, *p* < 0.001), and perineural invasion (χ^2^ = 21.1141, *p* < 0.001). However, no significant difference was detected between CORO1C expression and gender, age, location, differentiation, tumor size, preoperative CEA, preoperative CA199, and Ki67 (Table 1).

**TABLE 1:** Association between CORO1C expression and clinicopathological characteristics in CRC patients.

Characteristic	*n*	CORO1C(%)		Pearsonχ2	*P*
		High	Low or no		
Total	581	465 (63.35)	269 (36.65)		
Gender				1.0839	0.298
Male	348	226 (64.94)	122 (35.56)		
Female	233	161 (69.10)	72 (30.90)		
Age				0.5923	0.442
<60	212	137 (64.62)	75 (35.38)		
≥60	369	250 (67.75)	119 (32.25)		
Location				2.1214	0.548
Right	186	119 (63.98)	67 (36.02)		
Transverse	76	49 (64.47)	27 (35.53)		
Left	113	74 (65.49)	39 (34.51)		
Sigmoid	206	145 (70.39)	61 (29.61)		
Histological type				7.6419	0.006*
Adenocarcinoma	519	336 (64.74)	183 (35.26)		
Mucinous/SRCC	62	51 (82.26)	11 (17.74)		
Differentiation				1.6203	0.655
Well	154	98 (63.64)	56 (36.36)		
Moderate	312	215 (68.91)	97 (31.09)		
Poor	98	63 (64.29)	35 (35.71)		
Others	17	11 (64.71)	68 (35.29)		
T stage				1.2279	0.746
T1	41	27 (65.85)	14 (34.15)		
T2	60	39 (65.00)	21 (35.00)		
T3	198	127 (64.14)	71 (35.86)		
T4	282	194 (68.79)	88 (31.21)		
N stage				19.1615	<0.001*
N0	326	193 (59.20)	133 (40.80)		
N1	166	123 (74.10)	43 (25.90)		
N2	89	71 (79.78)	18 (25.90)		
M stage				9.5623	0.002*
M0	485	310 (63.92)	175 (36.08)		
M1	96	77 (80.21)	19 (19.79)		
AJCC stage				17.7192	0.001*
Ⅰ	62	35 (56.45)	27 (43.55)		
Ⅱ	197	114 (57.87)	83 (42.13)		
Ⅲ	231	173 (74.89)	58 (25.11)		
Ⅳ	91	65 (71.43)	26 (28.57)		
Venous invasion				22.1337	<0.001*
Negative	498	313 (62.85)	185 (37.15)		
Positive	83	74 (89.16)	9 (10.84)		
Perineural invasion				21.1141	<0.001*
Negative	504	318 (63.10)	186 (36.90)		
Positive	77	69 (89.61)	8 (10.39)		
Preoperative CEA, ng/ml				1.9482	0.378
≤5	257	166 (64.59)	91 (35.41)		
>5	283	196 (69.26)	87 (30.74)		
Unknown	41	25 (60.98)	16 (39.02)		
Preoperative CA199,ng/ml				2.6548	<0.265
≤37	259	164 (63.32)	95 (36.68)		
>37	279	195 (69.89)	84 (30.11)		
Unknown	43	28 (65.12)	15 (34.88)		
Ki67				0.4627	0.496
Negative	178	115 (64.61)	63 (35.39)		
Positive	403	272 (67.49)	131 (32.51)		

### High CORO1C Expression was Associated With Poor Prognosis in Colorectal Cancer

**FIGURE 3:**
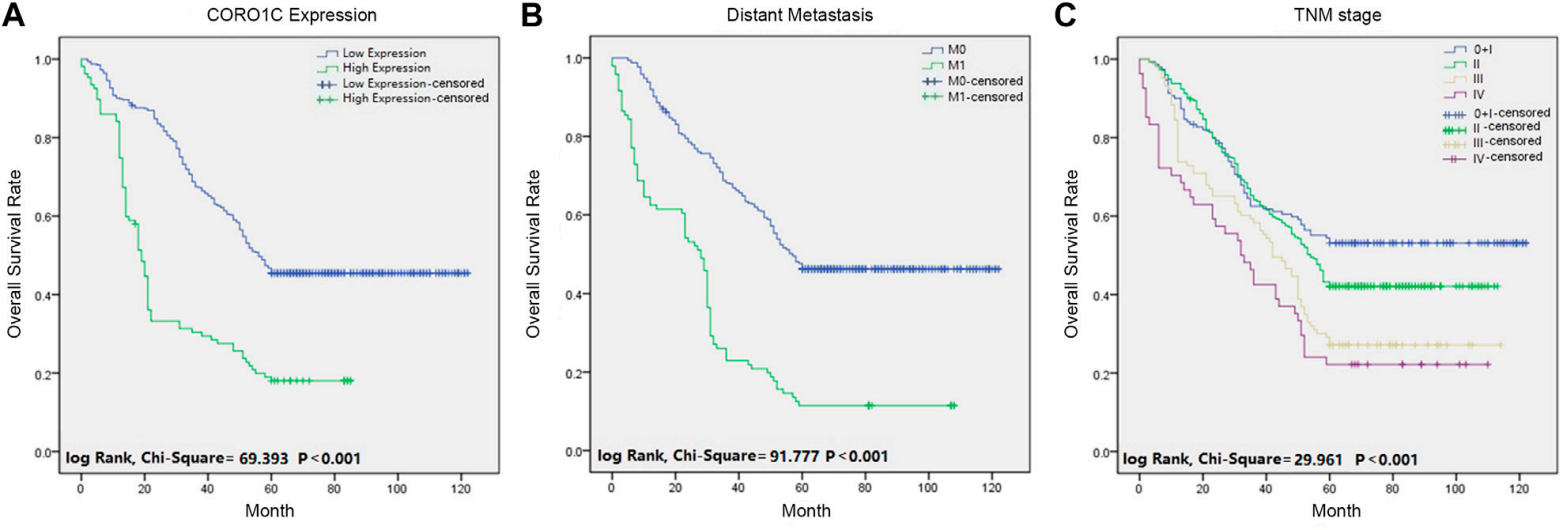
Survival curves of CRC patients using the Kaplan-Meier method and the log-rank test. **(A)** OS curves for patients with low CORO1C expression (blue line) and patients with high CORO1C expression (green line). **(B)** OS curves for distant metastases, M0 (blue line), and M1 (green line). **(C)** OS curves by TNM stage, TNM 0 and I (blue line), TNM II (green line), TNM III (gray line), and TNM IV (purple line).

Univariate analysis showed that the overall survival (OS) was significantly associated with CORO1C expression, differentiation, lymph node status, distant metastasis, TNM stage, venous invasion, perineural invasion, and preoperative CEA. Multivariate analysis indicated that only CORO1C expression, distant metastases, TNM stage, and venous invasion were independent prognostic factors for OS (Table 2). Moreover, Kaplan–Meier survival curves displayed that high CORO1C expression and increased distant metastasis exerted a negative effect on OS (Figure 3).

**TABLE 2:** Univariate and multivariate analysis of prognostic factors for overall survival in CRC.

	Univariate analysis			Multivariate analysis		
	HR	*p*-value	95%CI	HR	*p*-value	95%CI
CORO1C						
High vs. low or No	0.981	<0.001*	0.978−0.984	2.113	<0.001*	1.642−2.721
Age						
<60 vs. ≥60	1.151	0.338	0.864−1.533	—	—	—
Gender						
Male vs. female	1.150	0.350	0.858−1.540	—	—	—
Location						
Right vs. transverse vs. left vs. sigmoid	1.001	0.982	0.919−1.091	—	—	—
Histological type						
Adenocarcinoma vs. Mutinous/SRCC	0.981	0.892	0.744−1.294	—	—	—
Differentiation						
Well vs. moderate vs. poor vs. others	1.208	0.008*	1.051−1.389	—	—	—
TNM						
0 vs.Ⅰvs.Ⅱvs.Ⅲvs.Ⅳ	1.249	0.007*	1.063−1.467	1.178	0.007*	1.045−1.327
T stage						
T1 vs. T2 vs. T3 vs. T4	1.089	0.281	0.932−1.273	—	—	—
N stage						
N0 vs. N1 vs. N2 vs. N3	0.711	0.001*	0.582−0.870	—	—	—
M stage						
M0 vs. M1	2.243	<0.001*	1.502−3.350	3.305	<0.001*	2.529−4.320
Venous invasion						
Negative vs. positive	2.720	<0.001*	2.127−3.478	3.326	<0.001*	2.511−4.170
Perineural invasion						
Negative vs. positive	2.523	<0.001*	1.678−3.794	—	—	—
Preoperative CEA, ng/ml						
≤5 vs. >5 vs. unknown	1.025	0.824	0.827−1.270	—	—	—
Preoperative CA199,ng/ml						
≤37 vs. >37vs. unknown	0.955	0.674	0.771−1.184	—	—	—
Ki67						
Negative vs. positive	0.990	0.930	0.800−1.227	—	—	—

### Downregulation of CORO1C Inhibited Proliferation, Migration, and Invasion of CRC Cells *in Vitro* and *in Vivo*

**FIGURE 4:**
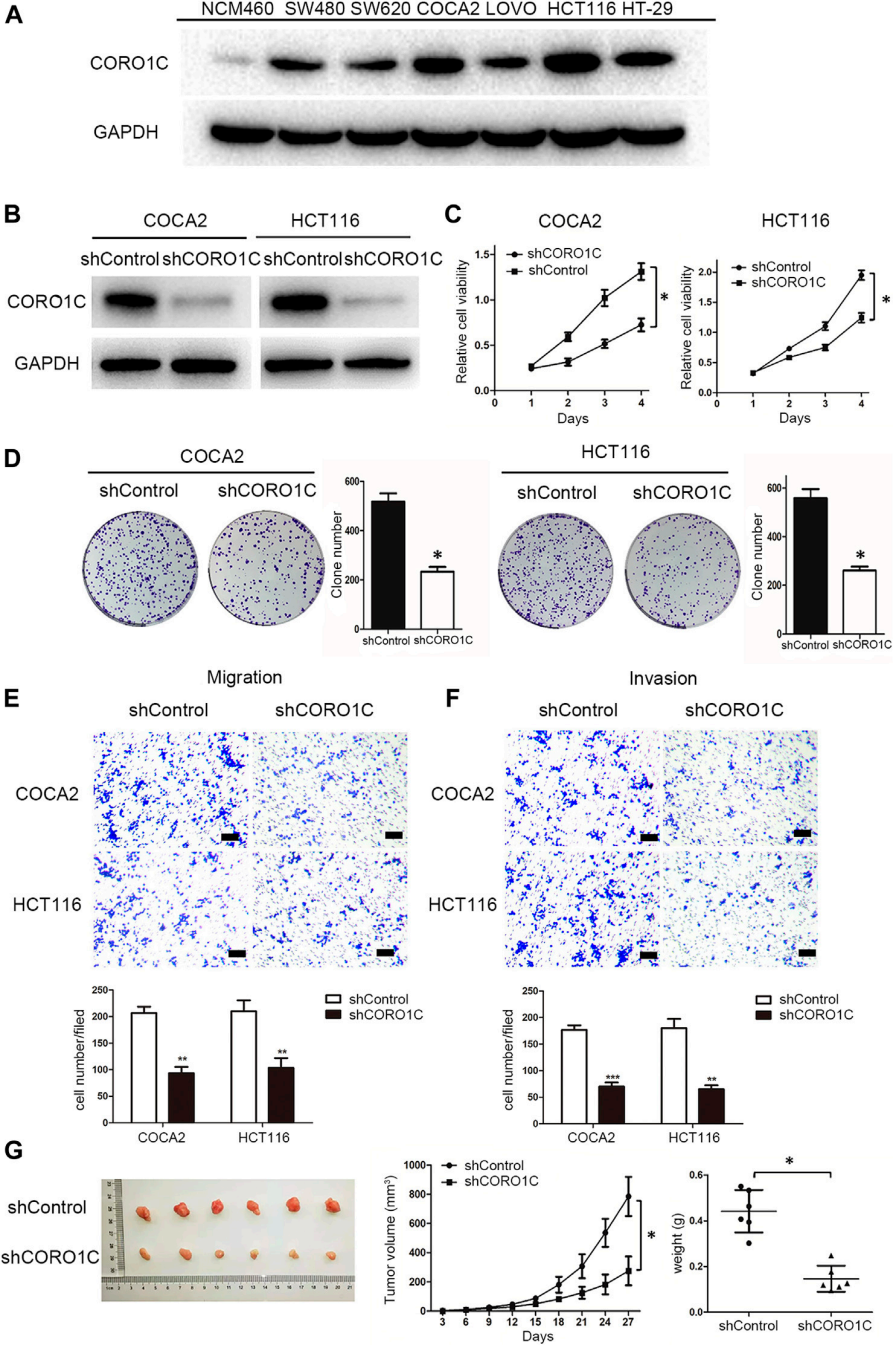
The effects of CORO1C knockdown on CRC growth and metastasis *in vitro* and *in vivo*. **(A)** Levels of CORO1C1 protein expression in CRC cell lines and normal colorectal epithelial cells (NCM460) determined by western blotting. **(B)** COCA2 and HCT116 cells showed a significant decrease in protein level after shCORO1C transfection. **(C)** CORO1C downregulation significantly inhibited the proliferation of both cell lines. **(D)** A significant decrease in cell anchorage-dependent growth was detected after CORO1C knockdown. **(E, F)** Decreased CORO1C expression impaired abilities of migration **(E)** and invasion **(F)** of CRC cells (scale bar, 150 μm). All quantitative data of *in vitro* assays were generated from three replicates **(G)**. The effects of CORO1C downregulation on the tumor growth in the xenograft mouse model (*n* = 6 mice/per group). *p < 0.05, **p < 0.01, ***p < 0.001.

We measured the levels of CORO1C expression in six CRC cell lines, namely SW480, SW620, COCA2, LOVO, HCT116, HT-29, and the normal colorectal epithelial cell line (NCM460) by western blotting. We found that CORO1C was highly expressed in COCA2 and HCT116 cells, and weakly expressed in SW620 and LOVO cells (Figure 4A). In the follow-up experiments, COCA2 and HCT116 cells were used to down-express CORO1C. SW620 and LOVO cells were used to over-express CORO1C.

To confirm the effects of CORO1C on CRC cells behaviors, shRNA against CORO1C was constructed and transfected into COCA2 and HCT116 cell lines. The significant downregulation of CORO1C after transfection was confirmed by Western blotting (Figure 4B). The knockdown of CORO1C in both cells suppressed cell proliferation (Figure 4C), clone formation (Figure 4D), migration (Figure 4E), and invasion (Figure 4F). A nude mouse xenograft model was constructed by HCT116 cells transfected with shRNA-CORO1C to confirm the role of CORO1C in CRC proliferation. The results showed that volumes (785.221 ± 134.681 mm^3^) and weights (0.442 ± 0.093 g) of tumors from the shCORO1C group (275.171 ± 98.854 mm^3^, 0.146 ± 0.057 g) were decreased than those from the control group, and CORO1C knockdown reduced the growth rate of CRC *in vivo* (Figure 4G).

### AKT Inhibition Reversed the Effects of CORO1C on Migration and Invasion in CRC Cells

**FIGURE 5:**
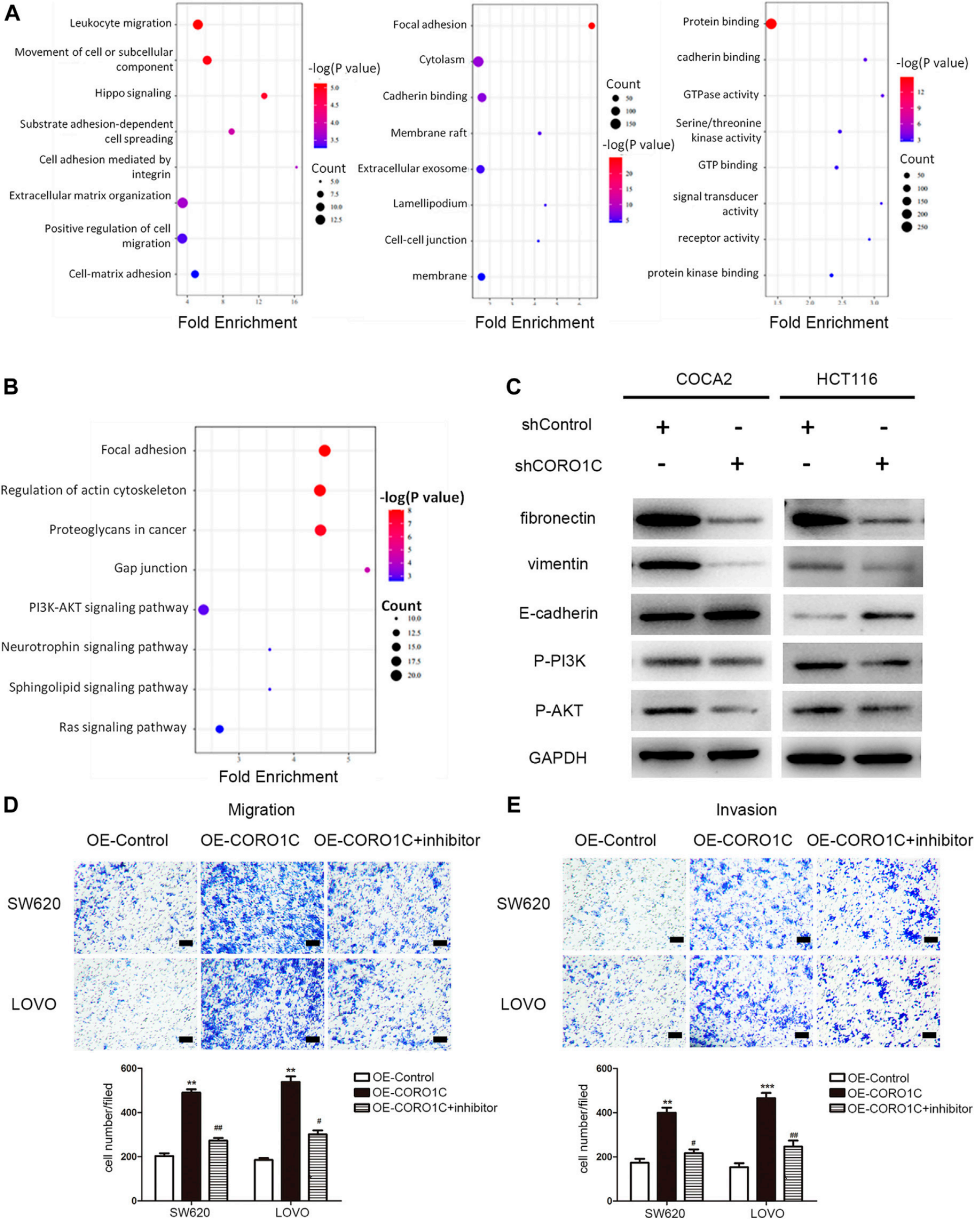
Underlying mechanism of CORO1C in CRC. **(A)** GO analysis of CORO1C-related genes in CRC. **(B)** GSEA of CORO1C-related genes in CRC. **(C)** Expression changes of EMT-related proteins and PI3K/AKT signaling pathway after CORO1C knockdown. **(D, E)** The effects of AKT inhibitor (Miltefosine) on the abilities of migration **(D)** and invasion **(E)** of CRC cells (scale bar, 150 μm). All quantitative data were generated from three replicates. *p < 0.05, **p < 0.01, ***p < 0.001 vs. OE-Control group, *#p <* 0.05, ##*p <* 0.01 vs. OE-CORO1C group.

As shown in Figure 5A, in the biological process, CROR1C-related genes were mainly enriched in leukocyte migration, movement of a cell or subcellular component, and cell adhesion. In the cellular component group, CROR1C-related genes were mainly enriched in focal adhesion, cadherin binding, and cell-cell junction. Regarding the molecular function, CROR1C-related genes were enriched in protein binding, cadherin binding, and GTP binding. The findings of GSEA confirmed that CORO1C was related to focal adhesion, regulation of actin cytoskeleton, gap junction, and PI3K/AKT signaling pathway (Figure 5B). In COCA2 and HCT116 cell lines transfected with shRNA-CORO1C, the expression of fibronectin and vimentin protein was decreased, and E-cadherin expression showed an opposite trend. In addition, CORO1C knockdown inhibited the phosphorylation of the PI3K/AKT signaling pathway (Figure 5C).

To further explore whether CORO1C1 facilitates migration and invasion of CRC cells via PI3K/AKT signaling pathway. CORO1C1 was overexpressed in SW620 and LOVO cell lines. We found that the effects of CORO1C1 were reversed by inhibition of AKT (Figure 5D–E). Taken together, the effects of CORO1C on migration and invasion might be mediated leastwise by PI3K/AKT signaling pathway in CRC cells.

## Discussion

In this study, we showed that a large number of proteins were connected with Trop2. The expression of these proteins was compared between primary CRC and secondary CRC, and CORO1C was markedly up-regulated in secondary CRC. Reportedly, CORO1C overexpression is related to poor prognosis in hepatocellular carcinoma, primary effusion lymphoma, and gastric cancer (Wu et al., 2010a; Luan et al., 2010; Cheng et al., 2019). To determine the role of CORO1C in CRC, we examined TMAs that included 734 samples of colorectal tissues and the relevant clinical data. With respect to Trop2 expression in colorectal tissues (Ohmachi et al., 2006), high CORO1C expression was detected more often in CRC tissues (66.61%) than in paracancerous tissues (52.14%). The CORO1C expression was associated with histological type, lymph node metastases, distant metastases, AJCC stage, venous invasion, and perineural invasion, indicating the aggressive characteristic of the cancer type. Furthermore, CRC patients with high CORO1C expression had a poor prognosis. Our findings suggested that CORO1C promotes CRC invasiveness and metastasis, i.e., poor disease-free survival and OS rates in patients.

In order to verify the influence of CORO1C on CRC, its expression was downregulated in COCA2 and HCT116 cells, which was inhibited clone formation, cell proliferation, migration, and invasion *in vitro*. The silencing also suppressed the tumor growth *in vivo*. As a target of several microRNAs, CORO1C promotes cell proliferation, invasion, and migration in non-small lung cancer, hepatocellular carcinoma, and triple-negative breast cancer (Wang et al., 2014; Han et al., 2020; Liao and Peng, 2020). It was demonstrated that knockdown of CORO1C dramatically suppressed cell viability, colony formation, mitosis, and metastasis and promotes apoptosis of gastric cancer cells (Cheng et al., 2019). These studies supported our findings that CORO1C plays a tumor-promoting role in CRC.

In order to elucidate the possible mechanisms, GO analysis and GSEA of CORO1C in CRC were performed. The CORO1C-related genes were classified into the biological process group, cellular component group, and molecular function group, indicating that CORO1C is associated with cell adhesion and migration. Cell invasion and metastasis require the involvement of the actin cytoskeleton, which is composed of filamentous actin (F-actin) (Jung et al., 2016). CORO1C is an actin-binding protein with three binding sites, directly regulating the activity of F-actin (Mikati et al., 2015). Several studies suggested a critical role of CORO1C in tumor metastasis. For example, it promotes gastric cancer metastasis via interaction with Arp2, MMP-9, and cathepsin K (Ren et al., 2012; Sun et al., 2014), increases matrix degradation and invasion and decreases adhesion and formation of invadopodia-like extensions in glioblastoma cells (Ziemann et al., 2013). The knockdown of CORO1C impairs cell polarity and cytoskeleton in hepatocellular carcinoma cells (Wang et al., 2013). GSEA demonstrated that CORO1C-related genes are enriched in PI3/AKT signaling pathway. Trop2 increases IGF-1R signaling-mediated AKT/β-catenin/Slug expression essential for cell survival and EMT (Shvartsur and Bonavida, 2015). As a binding protein of Trop2, CORO1C induces EMT through PI3/AKT pathway, which was confirmed by subsequent experiments. With CORO1C knockdown, the expression of EMT biomarkers such as fibronectin and vimentin was decreased, and that of E-cadherin was increased, suggesting that shCORO1C inhibits EMT in CRC cells. The downregulation of CORO1C also reduced the phosphorylation levels of the PI3K/AKT signaling pathway.

Our study nevertheless has some limitations. The other proteins besides CORO1C interacting with Trop2 on CRC were not explored. It's necessary if we want to fully understand the function of Trop2. In addition, the results will be more valuable if the detailed network around CORO1C and the downstream signaling pathways are investigated. These questions need to be researched in the future.

## Conclusion

In summary, we demonstrated that high CORO1C expression was associated with increased metastasis and poor prognosis. CORO1C induced EMT of CRC cells via PI3K/AKT signaling pathway and then promoted CRC invasion and metastasis. Therefore, CORO1C may be a potential prognostic biomarker and a therapeutic target for CRC patients.

## Data Availability

The original contributions presented in the study are included in the article/Supplementary Material, further inquiries can be directed to the corresponding authors.
