# Corrigendum: CORO1C is Associated with Poor Prognosis and Promotes Metastasis Through PI3K/AKT Pathway in Colorectal Cancer

**DOI:** 10.3389/fmolb.2021.727347

**Published:** 2021-08-30

**Authors:** Zongxia Wang, Lizhou Jia, Yushu sun, Chunli Li, Lingli Zhang, Xiangcheng Wang, Hao Chen

**Affiliations:** ^1^ Cancer Center, Bayannur Hospital, Bayannur, China; ^2^ Department of Pathology, Wannan Medical College, Wuhu, China; ^3^ Department of Oncology, Inner Mongolia Autonomous Region Cancer Hospital, Hohhot, China; ^4^ Department of Ophthalmology, Inner Mongolia Autonomous Region People’s Hospital, Hohhot, China; ^5^ Department of Nuclear Medicine, The Affiliated Hospital of Inner Mongolia Medical University, Hohhot, China; ^6^ Key Laboratory of Inner Mongolia Autonomous Region Molecular Imaging, Inner Mongolia Medical University, Hohhot, China; ^7^ Faculty of Medical Science, Jinan University, Guangzhou, China

**Keywords:** CORO1C, colorectal cancer, prognosis, metastasis, Akt

In the original article, there was a mistake in the caption for **Figure 4A** as published. The incorrect caption stated NCM460 cell lines are normal gastric epithelial cells, however, NCM460 cell lines are normal colorectal epithelial cells. The correct caption for **Figure 4** appears below. Additionally, there was a mistake in **Figure 1** as published. Figure 1C is immunofluorescence and **Figure 1D** is immunocomplex by Co-IP. OE-ControlTrop2 should appear on the top row and OE-Trop2 should appear on the bottom row in **Figure 1C**. Representative images for these parts contain small issues which have been corrected. The corrected **Figure 1** appears below.

**FIGURE 4**. The effects of CORO1C knockdown on CRC growth and metastasis *in vitro* and *in vivo*. **(A)** Levels of CORO1C1 protein expression in CRC cell lines and normal colorectal epithelial cells (NCM460) determined by western blotting. **(B)** COCA2 and HCT116 cells showed a significant decrease in protein level after shCORO1C transfection. **(C)** CORO1C downregulation significantly inhibited the proliferation of both cell lines. **(D)** A significant decrease in cell anchorage-dependent growth was detected after CORO1C knockdown. **(E, F)** Decreased CORO1C expression impaired abilities of migration **(E)** and invasion **(F)** of CRC cells (scale bar, 150 μm). All quantitative data of *in* *vitro* assays were generated from three replicates **(G)**. The effects of CORO1C downregulation on the tumor growth in the xenograft mouse model (*n* = 6 mice/per group). *p < 0.05, **p < 0.01, ***p < 0.001.

The authors apologize for this error and state that this does not change the scientific conclusions of the article in any way. The original article has been updated.

**FIGURE 1:**
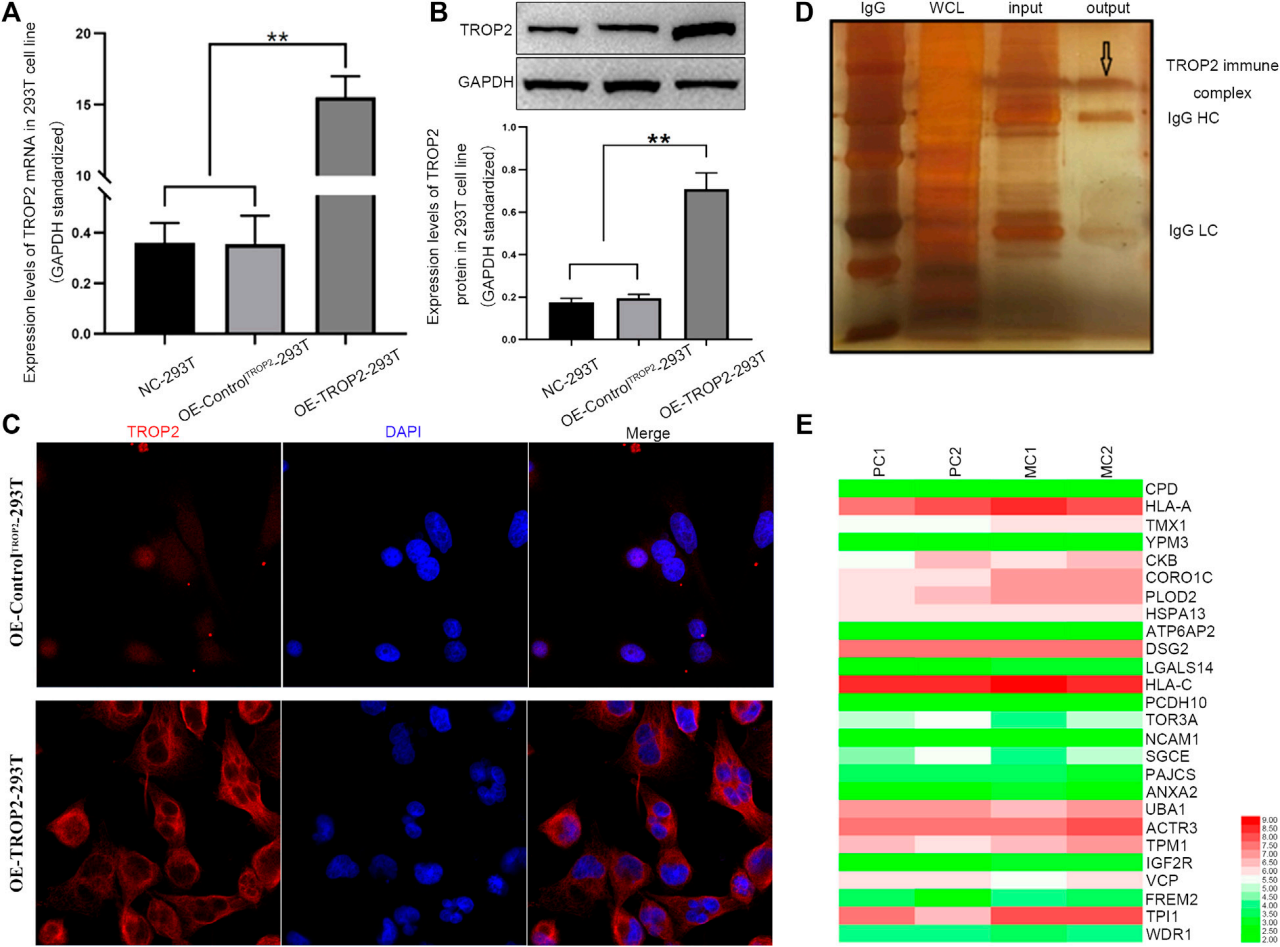
Associations between CORO1C, TROP2, and CRC metastasis. Overexpression of Trop2 in 293T cells was confirmed by **(A)** qRT-PCR, **(B)** Western blotting, and **(C)** Immunofluorescence. **(D)** Trop2 immunocomplex by Co-IP; **(E)** Heatmap of proteins interacting with Trop2 in PC and MC. The RNA-seq data were obtained from GSE28702. Red and green colors represent high and low gene expression, respectively. PC: primary CRC; MC: metastatic CRC, **p < 0.01.

