# Supplementary material for: CORO1C is Associated With Poor Prognosis and Promotes Metastasis Through PI3K/AKT Pathway in Colorectal Cancer
Source: Front Mol Biosci. 2021 Jun 10;8:682594. doi: 10.3389/fmolb.2021.682594 (PMC8223509; doi:10.3389/fmolb.2021.682594)
Supplement: Supplementary file 1 [file DataSheet1.docx]

**Supplementary Table 1. Differential intensity of top 10 proteins in TROP2 immune complexes measured by mass spectrometry.**

| Gene | Intensity T-01 | Intensity T-02 | Intensity T-03 |
| --- | --- | --- | --- |
| TROP2 | 1.11E+11 | 8.04E+10 | 9.92E+10 |
| CPD | 5.81E+08 | 4.22E+08 | 5.15E+08 |
| HLA-A | 5.30E+08 | 4.37E+08 | 4.38E+08 |
| PLOD2 | 1.52E+08 | 1.12E+08 | 1.28E+08 |
| TMX1 | 9.53E+07 | 1.54E+08 | 1.05E+08 |
| TPM3 | 8.20E+07 | 5.94E+07 | 7.79E+07 |
| CKB | 7.15E+07 | 4.52E+07 | 5.52E+07 |
| CORO1C | 4.37E+07 | 4.58E+07 | 4.54E+07 |
| HSPA13 | 3.50E+07 | 2.99E+07 | 3.16E+07 |
| LEPRE1 | 3.32E+07 | 2.19E+07 | 2.42E+07 |

| Gene | *P* value | Log FC |
| --- | --- | --- |
| PLOD2 | 0.003 | 2.270 |
| CORO1C | 0.011 | 1.490 |
| TPI1 | 0.049 | 1.260 |
| TOR3A | 0.053 | -1.260 |
| LGALS14 | 0.057 | 1.160 |
| WDR1 | 0.118 | -0.887 |
| NCAM1 | 0.123 | -4.960 |
| IGF2R | 0.123 | 0.786 |
| PCDH10 | 0.132 | -0.594 |
| TPM1 | 0.171 | 0.720 |

**Supplementary Table 2. Differential expressions of top 10 genes between primary CRC and** **secondary CRC.**
